# Intimate partner violence and termination of pregnancy: a cross-sectional study of married Bangladeshi women

**DOI:** 10.1186/s12978-015-0095-7

**Published:** 2015-11-05

**Authors:** Mosfequr Rahman

**Affiliations:** Department of Population Science and Human Resource Development, University of Rajshahi, Rajshahi, 6205 Bangladesh

**Keywords:** Intimate partner violence, Termination of pregnancy, Bangladesh

## Abstract

**Background:**

The prevalence of intimate partner violence (IPV) and its consequences on women’s reproductive health and pregnancy outcomes have been well documented. Bangladesh is burdened with the high prevalence of IPV and induced abortion/menstrual regulation. Understanding their association may benefit strategies to reduce termination of pregnancy (TOP). Therefore, this study assesses the association between experience of IPV and TOP among married Bangladeshi women age 15–49 years.

**Methods:**

This cross-sectional study is based on data from 10,146 married women of reproductive age from the Bangladesh Demographic Health Survey, 2007 (BDHS). A subset of interviews from currently married women, living with a husband and who had at least one pregnancy in the last 5 years (*n* = 1875) were extracted.

**Results:**

Results of this study showed that among the respondents, 31.4 % experienced physical and/or sexual IPV: 13.4 % experienced only sexual violence and 25.8 % experienced only physical violence. 21.0 % respondents ever had a TOP and 5.8 % had a TOP in last 5 years. Physical IPV was significantly associated with both TOP ever (OR = 1.36; 95 % CI: 1.05–1.77) and TOP in last 5 years (OR = 1.72; 95 % CI: 1.11–2.06).

**Conclusions:**

Prevention of intimate partner violence which was associated with pregnancy termination may reduce the high incidence of termination of pregnancies in Bangladesh.

## Background

Maternal mortality is unnecessarily high in developing countries—290 deaths per 100,000 live births compared to only 14 per 100,000 live births in developed countries—and more than 99 % of the annual global maternal deaths occur in developing countries [1]. Each pregnancy puts a woman at risk of death, but compared with women who have live births, those who have induced abortions, miscarriages or stillbirths have been found to be at a higher risk of maternal mortality [2–4]. Induced abortions in developing countries may be performed in unhygienic settings and carry a high risk of mortality [5, 6]. Moreover, it is found that induced abortions carry a higher risk of maternal mortality than miscarriages, while the risk of maternal death is higher among women who have stillbirths than those having live births [4]. Bangladesh, a country with poor socioeconomic conditions, has a moderate level of maternal mortality—194 deaths per 100,000 live births, for the period 2007–2010 [7] —especially considering its poorly managed and inadequate health infrastructure and high rate of non-institutional births [8]; only 15 % of births in Bangladesh take place in health facilities [9].

Intimate partner violence (IPV) against women is a pervasive public health concern and human rights violation of worldwide significance [10]. Globally, lifetime prevalence of IPV has been found to be between 10 and 52% [11] and even as high as 71% in some developing countries [12]. A recent report on violence against women in Bangladesh showed that 87 % currently married women had experienced IPV ever, and 77 % had reported IPV in the past 12 months [13]. Previous research indicated that women of childbearing age may be at a higher risk for IPV [14]. Recent comprehensive reviews have concluded that while national and international population-based studies found pregnant women no more likely or even at decreased risk of experiencing IPV than non-pregnant women, some hospital and clinic-based studies indicated an increased risk [15]. The estimated prevalence of violence against women during pregnancy ranges from 4 to 29 % in developing countries [16]. It is important to understand more about IPV and its association with termination of pregnancy (TOP) in different settings as the awareness of the prevalence of IPV and its negative sexual and reproductive health outcomes has widened [17].

How IPV relates to the death of neonates and infants is a critically important topic deserving of increased attention, particularly in South Asia where child mortality is relatively high and, notably, where girls suffer higher child mortality than boys, a disparity rarely seen globally [18]. A number of studies have been conducted indicating negative sexual and reproductive health outcomes associated with IPV including unwanted/unintended pregnancy [19–21], induced abortion [21–23], miscarriage [21, 24, 25], and fetal death [26–28]. The study conducted in Bangladesh, using data from the 2004 Bangladesh Demographic Health Survey (BDHS), found that 76 % of Bangladeshi women experienced IPV and that those women were more likely to report both unwanted pregnancy and miscarriage, induced abortion, or stillbirth [21]. However, that study was limited to men’s reports of IPV and did not measure the relationship between IPV and TOP directly. Therefore, this study, using women’s reports of IPV, explored the association between the TOP and IPV among married Bangladeshi women.

## Methods

**Fig. 1:**
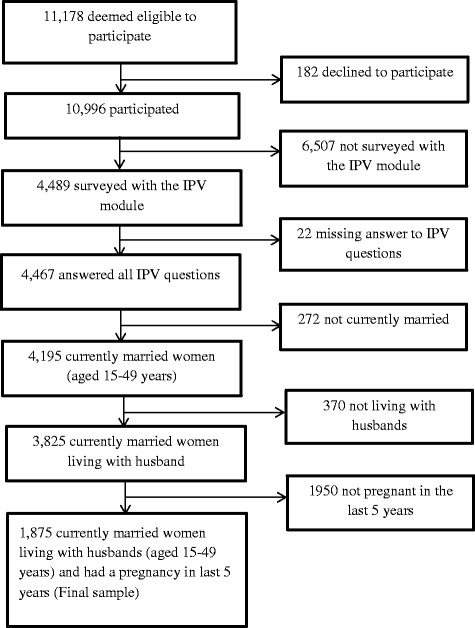
Selection of the sample

The study utilized data from the Bangladesh DHS (BDHS), which was carried out from March to August 2007 in collaboration with the Bangladesh National Institute for Population Research and Training (NIPORT) [29]. The data in this study was assessed from MEASURE DHS of the U.S. Agency for International Development (USAID). The BDHS sample was drawn from the total adult population of Bangladesh residing in private dwellings. A stratified, multistage cluster sample of 361 primary sampling units, 227 in rural areas and 134 in urban areas, was constructed. A total of 11,178 ever married women aged 15–49 were deemed eligible to participate in the survey, and 10,996 were interviewed (response rate was 98.4 %). The ORC Macro Institutional Review Board (Calverton, MD, USA) approved the data collection procedures of the BDHS. Data collection and management procedures are described in details elsewhere [29]. This study analyzed data from currently married women of age 15–49 years, living with their husbands and who had at least one pregnancy in the last 5 years. To obtain nationally representative estimates, weights from the Domestic Violence Module were used as sampling weights. For this study, the data set was restricted to 1875 married women who had a pregnancy during the last 5 years immediately preceding the date of the survey (for details on sample selection, see Fig. 1).

## Outcome measures

In the questionnaire of the 2007 Bangladesh Demographic Health Survey, lifetime history of miscarriage, induced abortion, and stillbirth was assessed for all women through a single item asking if they ever had a pregnancy that ended in miscarriage, ended due to an induced abortion or ‘menstrual regulation (MR)’ (a term used in Bangladesh to describe administration of legal abortive procedures by a clinician or clinical procedures to induce abortion), or ended in the stillbirth of a child. Respondents who reported ever having experienced a miscarriage, induced abortion, or stillbirth were asked whether such an event had occurred within the past 5 years. The term menstrual regulation and/or induced abortion is considered as the termination of pregnancy (TOP) in this study and dichotomized as had a TOP and had no TOP.

## Exposures

Women’s experience of IPV was the main exposure of interest in this study. In BDHS, questions on IPV in the Women’s and Men’s Questionnaires were administered to only one eligible respondent per household. Selecting only one person to receive the IPV questions protects the privacy of that person and helps to ensure that other respondents in the household are not aware of the types of questions that the selected respondent was asked. If there was more than one eligible female or male respondent in the household, the respondent was selected randomly through a specially designed simple selection procedure based on the Kish Grid [30]. The Kish grid provides a selection procedure by which eligible persons within the household stood an equal chance of being included in the survey [31]. Socioeconomic and cultural contexts within Bangladeshi society inhibit women from speaking about their experience of violence from their intimate partner. Many women may not disclose incidents of IPV because they fear further violence by the perpetrators or because of shame or embarrassment. Therefore, interviewers were instructed to collect information in complete privacy which was essential to ensure the security of the respondent and the interviewer as well. Informed consent was obtained from survey respondents at the beginning of the interview. The BDHS measured IPV with a shortened and modified version of the Conflict Tactics Scale (CTS) [32]. This study examined responses in the Women’s Questionnaires. Perpetration of IPV by the woman’s husband was assessed via 8 survey items. Women who reported that their husband engaged in any of the following behaviors were classified as having experienced physical IPV: i. pushing, shaking, or throwing an object; ii. slapping; iii. pulling hair or twisting an arm; iv. punching or hitting with a fist or something harmful; v. kicking or dragging; vi. choking or burning; or vii. threatening or attacking with a knife or gun. A positive answer of these questions indicated physical perpetration and questions (i) to (iv) indicated experience of minor physical violence and questions (v) to (vii) indicated experience of severe physical violence. Perpetration of sexual IPV was indicated by a woman’s positive response to an item asking whether she had been physically forced to have sexual intercourse even when she did not want to.

## Covariates

All sociodemographic variables were assessed via self-report, and the variables included age, age at first marriage, parity, education, place of residence, religion, and modern contraceptive use. The wealth index was constructed from data on household assets, including ownership of durable goods (such as televisions, refrigerator, mobile phone, bicycle, etc.) and dwelling characteristics (such as source of drinking water, sanitation facilities, and construction materials). To create the wealth index, each asset was assigned a weight (factor score) generated through principal component analysis, and the resulting asset scores were standardized in relation to a normal distribution, with a mean of 0 and standard deviation of 1 [33]. Each household was then assigned a score for each asset, and the scores were summed for each household; individuals were ranked according to the total score of the household in which they resided. The sample was then divided into quintiles with 1 = poorest and 5 = wealthiest 20 % of households.

## Ethical considerations

The 2007 BDHS data collection procedures were approved by the ORC Macro Institutional Review Board (Calverton, MD, USA). The protocol of the survey was reviewed and approved by the National Ethics Review Committee of the Bangladesh Ministry of Health and Family Welfare. Because the existence of a signed consent form can provide a risk in itself for the abused women, oral informed consent was obtained from respondents by interviewers [29]. Several specific protections based on WHO’s ethical and safety recommendations for research on domestic violence were built into the 2007 BDHS. These include: only administering the domestic violence module to one woman in each household; reiterating informed consent; ensuring privacy; emotional support for field staff; and developing quality assurance procedures.

## Statistical analysis

In this study, *χ*^2^ tests were used to assess the association between TOP and different forms of IPV among the respondents. The effect of different forms of IPV on TOP was estimated using logistic regression procedures. In all analyzes, the significance level was set at *P* < 0.05 (2-tailed). Multiple logistic regressions were used to estimate the net effects of different forms of IPV on TOP by controlling for theoretically relevant variables. Two fully adjusted models were used to analyze the appropriate binary value for each TOP variables (ever had termination of pregnancy and termination of pregnancy in last 5 years), with each model containing a different IPV predictor (physical and/or sexual IPV, physical IPV, sexual IPV, minor physical IPV and severe physical IPV). All covariates were entered simultaneously into the multiple regression models. Odds ratios (ORs) were estimated to assess the strength of the associations, and 95 % confidence intervals (CIs) were used for significance testing. The multicollinearity of the variables was checked by examining the variance inflation factors (VIF), which was <2.0. All statistical analyzes were conducted using Statistical Package for Social Sciences (SPSS) 20.0 for Windows (SPSS Inc., Chicago, IL) to accommodate the complex sampling design of the BDHS.

## Results

Table 1 displays mean or percentage distributions for the sociodemographic variables of interest. The mean age and the mean age at first marriage of the respondents was 27.19 years and 15.59 years, respectively. The majority of the respondents were from rural areas (63.7 %) and also Muslim (90.6 %). Almost thirty percent (29.3 %) of women had no education and nearly three percent (2.4 %) respondents were from the richest household.

**Table 1:** Sociodemographic characteristics of the respondents

Characteristics	Number	Percentage/Mean ± SD
Age (in years)	1875	26.16 ± 6.19
Age at first marriage	1875	15.59 ± 2.75
Place of residence		
Rural	1195	63.7
Urban	680	36.3
Respondents education		
No education	549	29.3
Primary	576	30.7
Secondary	603	32.2
Higher	147	7.8
Wealth index		
Poorest	420	22.4
Poorer	415	22.1
Middle	329	17.6
Richer	309	16.5
Richest	402	21.4
Religion		
Non-Muslims	176	9.4
Muslims	1699	90.6
Wanted last birth		
Wanted then	1280	68.3
Wanted later	289	15.4
Wanted no more	306	16.3

Table 2 presents obstetrical factors and different forms of IPV experienced by respondents. More than twenty percent (21.0 %) respondents ever had a TOP and almost six percent (5.8 %) had TOP in the last 5 years. Almost one in every three women (31.4 %) experienced physical and/or sexual IPV while half of the respondents (50.1 %) experienced any form of minor physical violence. Moreover, nearly fifteen percent (13.4 %) of women experienced sexual violence from their husbands.

**Table 2:** Obstetrical factor and experience of intimate partner violence (IPV) among the respondents

Characteristics	Number	Percentage/Mean ± SD
Children ever born (CEB)	1875	2.79 ± 1.76
Ever used modern contraceptives		
No	323	17.2
Yes	1552	82.8
Ever had terminated pregnancy	394	21.0
Terminated pregnancy in last 5 years	109	5.8
Experience of physical and/or sexual IPV	589	31.4
Experience of physical IPV	484	25.8
Experience of sexual IPV	251	13.4
Experience of minor physical IPV	940	50.1
Experience of severe physical IPV	300	16.0

Table 3 presents the association between TOP and experience of IPV among respondents. Women who experienced physical IPV, sexual IPV, minor physical IPV and severe physical IPV were significantly more likely to report TOP ever (23.4 % vs 20.1 %; *P* = 0.032), (21.1 % vs 16.2 %; *P* = 0.038), minor physical IPV (24.3 % vs 17.7 %; *P* = 0.000) and severe physical IPV (27.0 % vs 19.8 %; *P* = 0.005), respectively than women who did not experience physical IPV. Women who experienced minor physical IPV (6.7 % vs 4.9 %; *P* = 0.033) and severe physical IPV (8.5 % vs. 5.3 %; *P* = 0.030) were significantly more likely to report TOP in the last 5 years than women who had not experienced minor physical IPV and severe physical IPV, respectively.

**Table 3:** Association between different forms of IPV and TOP among respondents

Experience of IPV	Ever had TOP (%, 95 % CI)	TOP in last 5 years (%, 95 % CI)
Physical and or sexual IPV		
Yes	22.8 (19.6–26.3)	6.4 (4.5–9.0)
No	20.1 (18.0–20.4)	5.4 (4.2–7.0)
*P*-value	0.186	0.441
Physical IPV		
Yes	23.4 (18.1–22.3)	6.6 (4.5–9.6)
No	20.1 (18.1–27.4)	5.5 (4.3–7.0)
*P*-value	0.032	0.403
Sexual IPV		
Yes	21.1 (16.5–26.6)	4.5 (2.4–8.3)
No	16.2 (14.1–203)	6.0 (4.8–7.4)
*P*-value	0.038	0.386
Minor physical IPV		
Yes	24.3 (21.6–27.1)	6.7 (5.1–8.7)
No	17.7 (15.4–20.2)	4.9 (3.5–6.7)
*P*-value	0.000	0.033
Severe physical IPV		
Yes	27.0 (22.3–32.3)	8.5 (5.6–12.6)
No	19.8 (17.9–21.9)	5.3 (4.2–6.7)
*P*-value	0.005	0.030

Table 4 provides a logistic regression analysis to estimate the net effects of different forms of IPV on pregnancy termination by controlling for theoretically relevant variables. Women who experienced physical and/or sexual IPV are 1.29 times significantly more likely to have a TOP ever. However, the experience of physical IPV was significantly positively associated with TOP ever (AOR = 1.36; 95 % CI: 1.05–1.77) and TOP in the last 5 years (AOR = 1.72; 95 % CI: 1.11–2.06). No significant association is found between the experience of sexual IPV and any forms of TOP. Women who experienced minor physical IPV are 1.47 times and 1.84 times significantly more likely to have a TOP ever and TOP in the last 5 years, respectively, than women who had not experienced minor physical IPV, while the experience of severe physical IPV was significantly associated with ever having had a TOP.

**Table 4:** Results of logistic regression analysis to predict the association between TOP and different forms of IPV

Experience of IPV	Ever had TOP		TOP in last 5 years	
	Crude OR (95 % CI)	Adjusted OR (95 % CI)	Crude OR (95 % CI)	Adjusted OR (95 % CI)
Physical and/or sexual IPV	1.35*** (1.09–1.81)	1.29** (1.01–1.65)	1.73** (1.29–3.41)	1.61 (0.99–2.60)
Physical IPV	1.44*** (1.16–2.01)	1.36*** (1.05–1.77)	1.80*** (1.23–3.21)	1.72** (1.11–2.06)
Sexual IPV	1.01 (0.73–1.40)	1.03 (0.74–1.44)	0.73 (0.36–1.48)	0.83 (0.41–1.71)
Minor physical IPV	1.49*** (1.19–1.87)	1.47** (1.16–1.87)	1.40** (1.08–2.17)	1.84** (1.16–2.93)
Severe physical IPV	1.50** (1.13–1.98)	1.45*** (1.08–1.94)	1.66* (1.04–2.76)	2.20 (1.28–3.77)

***p < 0.001, **p < 0.01, *p < 0.05

*OR* odds ratio, *CI* confidence interval. Models were adjusted for age, age at first marriage, education, place of residence, wealth index, religion, wanted last child, children ever born and ever used modern contraceptive method

## Discussion

A high percentage of women experience IPV during pregnancy, which may render them vulnerable to adverse pregnancy outcomes, including abortion and miscarriage [21–25, 27]. Considering the high pregnancy rate and the prevalence of violence during pregnancy which ranges from 4 to 29 % in developing countries, it clearly reflects that violence during pregnancy is a major public health problem [16]. Consistent with previous research around the world [19, 23, 34], including Bangladesh [21], this study’s findings demonstrate a significant association between IPV and TOP among Bangladeshi women. The association between IPV and TOP found in this study bolsters the previous findings that being in an abusive relationship with intimate partner may affect women’s reproductive decision making which can result in TOP [34]. A plausible reason may be that women in abusive relationships may have low autonomy over their sexual lives and therefore can have more unwanted pregnancies [21], which in turn may increase the number of pregnancy terminations. IPV may increase the likelihood of unintended pregnancy by affecting pre-conception and post-conception desire for pregnancy, pregnancy preparations and adaptations to pregnancy [35] and, therefore, may lead to a higher rate of terminations [36]. Another possible explanation could be that in an abusive relationship, the husband may not want the child and directly forces his wife to terminate the pregnancy or indirectly may create situations which in turn influence the woman to take decision to terminate. Although, TOP may be the woman’s choice, alternative options for a woman may also be limited in an abusive relationship. These findings are important for health care workers who provide prenatal and post-abortion care; as they should consider the role of IPV on a patient’s current situation.

This study adds growing evidence that women who have experienced any form of physical IPV by their husbands were more likely to report terminated pregnancies than non-abused women. Women in a violent relationship may be more likely to obtain a TOP because of her reluctance to bring a child into a setting of violence. In such a situation, a woman may feel less prepared (emotionally, socially or financially) to take care of a child which may contribute to her decision to terminate a pregnancy. Theorists have proposed that induced abortion/TOP is a method by which an abused women may regain her reproductive health control [21]. Furthermore, a study of the US Agency for International Development (USAID) showed that TOP is associated with reduced maternal mortality via improved access to family planning [37]. However, the use of suitable family planning methods may not be accessible for women in violent relationships. Therefore, family planning programs in Bangladesh need to consider the role of IPV in women’s reproductive health. The prevalence of IPV reported by Bangladeshi women is very high- approximately 87 % of the women experienced violence ever from their husbands [13]. Given the adverse outcomes of IPV, this high prevalence has alarming consequences for the health and wellbeing of Bangladeshi women.

When the association between having a TOP and sexual violence alone is taken into account, it appears that very few people reported sexual violence and that there was no apparent association with pregnancy outcomes. One of the possible explanations could be the wide acceptance of sexual violence within marriage in Bangladeshi society. In support of this explanation, Garcia-Moreno et al. [38] showed in a WHO multi-country study that there is a higher acceptance of sexual violence compared to physical violence. Moreover, in accordance with an earlier study [39], low rates of sexual violence may indicate the under-reporting of sexual violence in Bangladesh. The BDHS includes a single question on sexual violence which used a narrow measure (i.e. forced sex). This might be a reason for potentially inhibiting a positive response to the sexual violence question. Further research with more comprehensive sexual IPV-related questions are needed to examine the effect of sexual IPV on TOP.

The current study has several limitations. First, the cross-sectional nature of the study does not allow for assessment of the chronology of the relationship between IPV and TOP or inferences regarding causality. Another limitation is underreporting and recall bias of having experienced violence and birth outcomes due to the sensitivity of the topic and retrospective nature of the survey. It must be considered in interpreting the present findings that the cases of induced abortion might be underreported as it is strictly restricted according to Bangladeshi law. However, menstrual regulation up to 10 weeks after the last menstrual period was introduced in the national family planning program in 1979 as an effort to reduce unsafe abortions [40]. While the BDHS attempts to facilitate the reporting of abortions by including the event ‘menstrual regulation’, stigma may have resulted in abortions being misreported as other forms of termination, for example miscarriage, to avoid stigmatization [21]. Despite these limitations, the main strength of this study includes the fact that it was based on a nationally representative sample; it used pre-tested well-designed questionnaires together with trained and educated interviewers for data collection with good reliability.

To protect women from violence and prevent unwarranted termination of pregnancy, healthcare providers need to intervene by screening for and dealing with violence, and greater accessibility to health care and use of contraceptives are also needed. Empowering women by improving education and social support would also enhance their self-esteem and better equip them to take on challenging circumstances [41]. Additional efforts by government and non-government organizations are needed to protect women from IPV and to promote more effective reproductive health programs that provide physical and emotional support for abused women. Establishing women’s equal rights and improving status through education and employment is also essential. Moreover, the existing law for protecting women from abuse must be strictly implemented. Finally, the involvement of husbands, the perpetrators of violence by education or counseling, is critical to reducing IPV.

## Conclusions

Addressing factors that contribute to TOP is an important step in reducing the reproductive health burden of women. The findings of this study confirmed that women who had been exposed to IPV were more likely to report a TOP than those who were not. This finding may reflect the inadequate social support for maternal health in Bangladesh. Proper screening of IPV is needed to provide counseling and other social support for women in crisis. In this regard, gynecological and obstetric services may be key intervention points to screen IPV. Moreover, midwives may play an important role by providing emotional, psychological or even material support to the abusive women at the pre or post-abortion care. Therefore, comprehensive and culturally sensitive IPV training and interventions are needed for the midwives to raise awareness about IPV, perceived responsibility and self-confidence in identifying and assisting IPV sufferers. Future longitudinal research to determine the magnitude of the relationship between IPV and TOP, the mechanisms through which IPV leads to TOP, and long-term effects of IPV and TOP on women is needed to provide clearer understanding of these issues.
